# Purkinje Fibers in Canine False Tendons: New Anatomical and Electrophysiological Findings

**DOI:** 10.1155/2020/8156928

**Published:** 2020-06-15

**Authors:** Ming Liang, Zulu Wang, Yi Li, Yanchun Liang, Yuji Zhang, Jingjing Rong, Yang Lv, Qi Zhang, Guitang Yang, Mingyu Sun, Junqi Wang, Sainan Li, Xunzhang Wang, Yaling Han

**Affiliations:** ^1^Department of Cardiology, General Hospital of Northern Theater Command, Shenyang 110840, China; ^2^Heart Rhythm Center, The Heart Institute, Cedars Sinai Medical Center, Los Angeles, CA 90048, USA

## Abstract

**Introduction:**

Purkinje system and false tendons (FTs) are related to ventricular arrhythmia, but the association between Purkinje fibers and FTs is not clear. This study investigated the associations of anatomical and electrophysiological characteristics between Purkinje fibers and FTs.

**Methods and Results:**

We optimized the protocol of Lugol's iodine solution staining of Purkinje fibers to study the anatomical structure and originated a novel electrophysiological mapping method, named the direct visual mapping (DVM) method, to study the electrophysiological characteristics. By using the above-mentioned innovations in 12 dogs, we found the following. (1) There was no Purkinje fiber found 0.5 cm–1.0 cm below the valve annulus or on the leaflets or chordae tendineae of the mitral valve or adjacent to the top 1/3 of the papillary muscle. (2) Purkinje fibers existed in all FTs, including smaller and tiny FTs. (3) The Purkinje fibers contained in the FTs extended from the proximal to the distal end, and their electrophysiological characteristics were similar to the fibers on the endocardium, including anterograde, retrograde, and decremental conduction and automaticity.

**Conclusions:**

Purkinje fibers are commonly found in FTs. The electrophysiological characteristics of the Purkinje fibers contained in FTs are similar to the fibers on the endocardium. FTs might have an anatomical and electrophysiological basis for ventricular arrhythmia.

## 1. Introduction

The Purkinje network is a specialized conduction system within the heart responsible for the electrical conduction of the ventricles, and it is also involved in the mechanism of certain ventricular tachyarrhythmias (named Purkinje-related arrhythmias), including monomorphic ventricular tachycardia (VT), polymorphic VT, and ventricular fibrillation (VF) [1–3]. The false tendon (FT) is a common intraventricular anatomical variation. It refers to a fibroid or fibromuscular structure that exists in the ventricle besides the normal connection of papillary muscle and mitral or tricuspid valve. Many clinical studies have shown that FTs are closely related to ventricular arrhythmias [4–7]. Idiopathic left ventricular tachycardia (ILVT) is a common reentrant ventricular arrhythmia originating from the left ventricle. It has been reported that its anatomic substrate is closely related to FT [8, 9]. FTs are also related to the occurrence of ventricular premature beats [10, 11]. Does FTs have the anatomical and electrophysiological basis for ventricular arrhythmia? In some previous studies, Purkinje cells were found in FTs [6, 12]. However, is the existence of Purkinje fiber in FTs a coincidence or a common phenomenon? Whether these Purkinje fibers have electrical conduction properties and what roles these Purkinje fibers play in ventricular arrhythmias has not been well investigated.

## 2. Materials and Methods

### 2.1. Heart Isolation and Preparation

All the protocols for animal experiments in this research were approved by the Institutional Animal Care and Use Committee (IACUC) of the General Hospital of Northern Theater Command. All procedures in animal experiments were in full compliance with recommendations on animal studies per the Helsinki Declaration of the World Medical Association and European (2010/63/EU) guidelines for the care and use of laboratory animals.

Twelve mongrel dogs (21.5 ± 2.5 kg) were used in this research. These dogs were anesthetized with midazolam (0.5 mg/kg IM) and then put to death by injecting air (100–150 ml) into their femoral vein. The hearts were harvested via a medial sternotomy approach immediately after death. According to different experimental purposes, the hearts were prepared and processed as soon as possible. The endocardium of six hearts was stained by Lugol's solution to observe the anatomical structure of Purkinje fibers. Another six hearts were used to observe the electrophysiological characteristics of the Purkinje system by a visual mapping method. The heart preparation method for Lugol's solution staining and visual mapping was as follows. First, the in vitro heart was incised from the mitral valve annulus to the apex along the left free-wall ventricle, and the aortic ring was incised to fully expose the left ventricular anterior and posterior papillary muscles. Then, the endocardium was relatively flattened, and the residual blood on the endocardium was cleared using a cotton swab.

### 2.2. Lugol's Solution Staining

Lugol's solution was prepared in advance (shorter than one week) by dissolving *I*_2_ (4%, *w*/*v*) and *KI* (4%, *w*/*v*) in deionized water under mild condition, and it was protected from light. Lugol's solution was sprayed evenly on the endocardium of a prepared heart and left to soak for 0.5–2 minutes to stain the Purkinje fibers. The staining process was repeated if the color faded or disappeared. A stereoscopic microscope (anatomical lens) was used to reveal small or complex anatomical structures.

### 2.3. Histological Staining

The hearts were fixed in 10% formalin, embedded with paraffin, and sectioned into 5 *μ*m slices. The tissue sections were stained with hematoxylin-eosin (HE) staining, Masson's stain, periodic acid Schiff (PAS) staining, and connexin 40 immunohistochemical staining.

### 2.4. Direct Visual Mapping Method

The prepared heart was submerged in a warmed (37°C) lactated Ringer's solution. Two 20-electrode high density catheters (1 mm electrode; 1 mm spacing) were placed closely on the region of interest under direct vision, such as the branch of Purkinje fibers, anterior or posterior papillary muscles, and FTs. Then, electrophysiological study and mapping, including programmed stimulation, activation mapping, and pace mapping, were performed in vitro. The heart was submerged in cold (<10°C) lactated Ringer's solution for five seconds, if sustained arrhythmia (such as VT or VF) was not pace terminable or automatic electrical activity needed to be stopped. This endocardial mapping method is termed direct visual mapping (DVM) method.

## 3. Results

### 3.1. The Anatomical Characteristics of Purkinje Fibers

**Figure 1:**
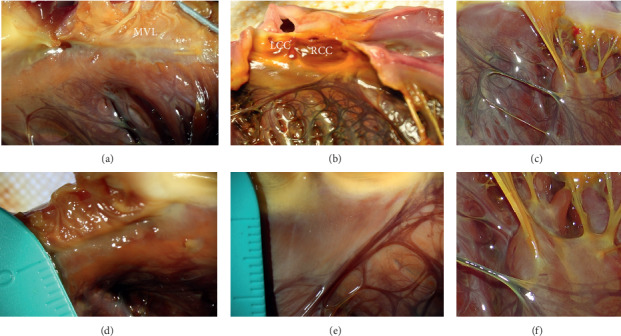
The distribution of Purkinje fibers in the left ventricular endocardium. (a, d) There was no Purkinje fiber branch found within 0.5 cm below the mitral valve; (b, e) showed there was no Purkinje fiber branch found within 0.8 cm below the aortic valve; (c, f) there was no Purkinje fiber branch found on the tip of the papillary muscles. MVL, mitral valve leaflet; LCC, left coronary cusp; RCC, right coronary cusp.

The left His-Purkinje system originated from underneath the border between the noncoronary sinus and right coronary sinus. It gave rise to a left and right bundle branch. There was no Purkinje fiber branch found 0.5 cm–1.0 cm below the valve annulus, including the aortic valve and mitral valve, except the left His bundle between the noncoronary sinus and right coronary sinus. There was no Purkinje fiber branch on the leaflets or chordae tendineae of the mitral valve. The major branches of the left anterior or posterior branches spread to FTs which connected to the side walls of papillary muscles. However, no Purkinje fiber branch was found adjacent to the top 1/3 of the papillary muscle (Figure 1).

### 3.2. The Anatomical Characteristics of FTs

**Figure 2:**
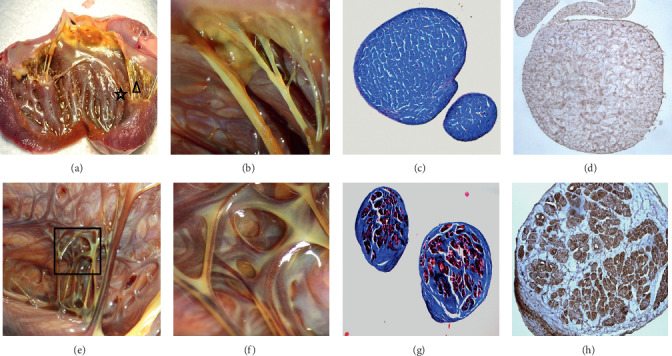
The distribution characteristics of Purkinje fibers on chordae tendineae (true tendons) and FTs. (a) Lugol's liquid staining in a dog's endocardium: Purkinje fibers were dyed darker. (b, c, and d) No Purkinje fibers contained in chordae tendineae which connected to the mitral valve. (b) The enlarged triangular region of (a). (c) Masson's staining of chordae tendineae (×100). (d) Connexin 40 immunohistochemical staining of chordae tendineae (×100). (e, f, g, and h) All of the FTs contain Purkinje fibers. (e) The enlarged star region of (a) (×5). (f) The enlarged rectangular area region of (e) (g) Masson's staining of FTs (×100). (h) Connexin 40 immunohistochemical staining of FTs (×100).

**Figure 3:**
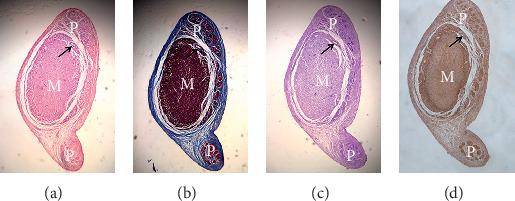
Purkinje fibers, typical working myocardium, and vasa vasorum in the larger FTs (×50). (a) HE staining; (b) Masson's staining; (c) PAS staining; (d) connexin 40 immunohistochemical staining. M, working myocardium; P, Purkinje fibers; black arrow, vasa vasorum.

FTs were found on the endocardium in all 12 dogs and the length varied. In all, 19 FTs were longer than 1 cm (2.4 ± 1.03 cm on the average; one to three FTs in each of the 12 dogs). In addition, there were 66 smaller FTs ranging from 0.5 to 1 cm in length (two to eight FTs in each of the 12 dogs), and tiny FTs (less than 0.5 cm) were more prevalent in each dog. Among the 19 longer FTs, five connected to anterior papillary muscles, 12 connected to posterior papillary muscles, and the remaining two did not connect to any papillary muscle. All of the FTs contain Purkinje fibers, including smaller and tiny FTs. The Purkinje fibers contained in the FTs extend from the proximal to the distal end and connected the endocardial Purkinje fibers to the papillary muscles or adjacent endocardial Purkinje fibers (Figure 2). Interestingly, there existed typical working myocardium and vasa vasorum in the larger FTs (Figure 3). On the contrary, there was no Purkinje fiber or myocardium contained in the chordae tendineae (true tendons), which connected the papillary muscles to the mitral valve leaflet (Figure 2).

### 3.3. Electrophysiological Characteristics of Purkinje Fibers on the FTs

By using the DVM method, the electrical activity of Purkinje fibers was recorded on the ventricular endocardium of harvested hearts by pacing the left ventricle or recording the automatic electrical activity. The electrical activation of the harvested heart could be recorded in 38 min∼80 min (56.5 ± 15.1 min).

**Figure 4:**
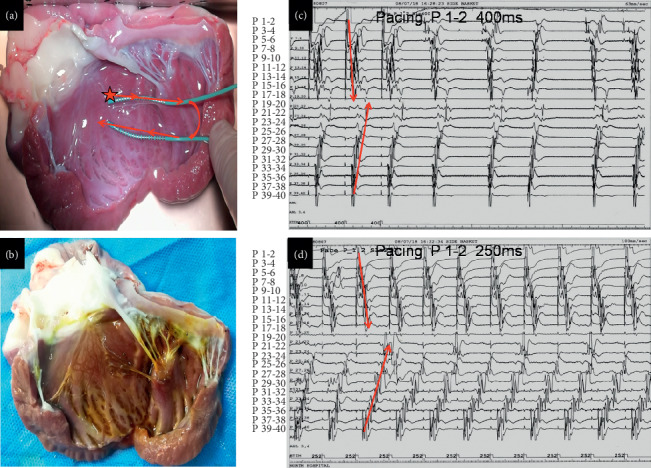
Electrophysiologic mapping of the endocardium using the DVM method. (a) Two 20-electrode mapping catheters were positioned at the LV septal endocardium from the upper septum to the apex. One mapping catheter (P1–P20) was stuck on one of the FTs, and the other one (P21–P40) was placed at the adjacent Purkinje network. Pacing point is located at the proximal end of FT (red star). (b) Lugol's liquid staining in the dog's endocardium: Purkinje fibers were dyed darker. (c) When pacing from the proximal FTs, at the cycle length of 400 ms, the conduction direction of Purkinje fibers contained in the FT was from the proximal to the distal end, and the conduction direction of Purkinje fibers on the apical endocardium was from the distal to the proximal end. After stopping the pacing, Purkinje fiber automaticity from FTs (the four to eight beats) was observed. (d) When pacing at a cycle length of 250 ms, decremental conduction of Purkinje fibers occurred, and the activation sequences of Purkinje fibers were the same as that of 400 ms pacing (c).

**Figure 5:**
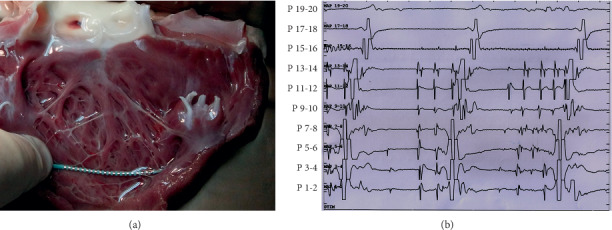
Multiple potentials were recorded in the overlapping area of FTs. (a) A 20-eletrode mapping catheter was positioned at the overlapping area of FTs; (b) multiple rows of Purkinje potentials were recorded.

The electrophysiological characteristics of Purkinje fibers on FTs were similar to fibers on endocardium, including bidirectional conduction and automaticity (Figure 4). Decremental conduction was also found in Purkinje fibers on FTs (Figure 4), especially during mechanical injury by slight traction. Multiple rows of Purkinje potentials were recorded in the overlapping area of FTs (Figure 5).

## 4. Discussion

### 4.1. Major Findings

Purkinje fibers are widely distributed in FTs, which connected the endocardial Purkinje fibers to the Purkinje fibers of papillary muscles or adjacent endocardium. The electrophysiological characteristics of Purkinje fibers contained in FT were similar to the Purkinje fibers on endocardium.

### 4.2. The Anatomical Distribution of the Purkinje System in the Left Ventricular Endocardium

The distribution of Purkinje fibers in the endocardium has certain characteristics. In this study, although Purkinje fibers are widely distributed in the endocardium, there is no Purkinje fiber branch under the mitral valve and the top 1/3 of the papillary muscle. There is no Purkinje fiber branch in the left ventricular outflow tract under the aortic valve except the left His bundle between the noncoronary sinus and right coronary sinus (Figure 1). These characteristics are very important to analyze the mechanisms of ventricular arrhythmias. For example, when mapping the premature ventricular complexes (PVCs) originating from the area of RCC, high frequency potentials are commonly recorded adjacent to the RCC in front of the QRS waves of PVCs. The potentials should not be considered as Purkinje fiber potential [13, 14].

### 4.3. The Anatomical and Electrophysiological Characteristics of FTs

FTs are single or multiple, thin, fibrous, or fibromuscular structures that traverse the cavity of the left ventricle and have no connection with the valvular cusps. FTs are very common in canine endocardium. It has been reported that FTs contain fibrous tissues, myocardial fibers, Purkinje fibers, and blood vessels. Purkinje cells were not observed in the specimens studied [7, 15].

We found that Purkinje fibers were commonly found in FTs, including smaller and tiny FTs. The Purkinje fibers contained in the FTs extends from the proximal to the distal end (Figure 2). The electrophysiological characteristics of Purkinje fibers contained in the FTs were similar to the fibers on the endocardium including bidirectional conduction, decremental conduction, and automaticity (Figures 4 and 5). Different FT components are not the same: some contain collagen fiber and Purkinje fiber, and some also contain myocardial fibers and nutrient vessels (Figure 3).

We speculate that the correlation between FTs and the Purkinje fiber system might provide anatomical and electrophysiological basis for FTs participating in ventricular arrhythmia. When the Purkinje fiber system (including FTs) is affected by mechanical traction, ischemia, hypoxia, or other factors, its electrophysiological characteristics might change accordingly, resulting in ventricular arrhythmias.

### 4.4. Clinical Implication

The reported prevalence of FTs in patients referred for echocardiography has varied widely between 0.8% and 61% in children and between 0.3 and 71% in adults [4–7]. Some studies reported that FTs might play important roles in ventricular arrhythmias, such as idiopathic fascicular VT or VT in structural heart disease, especially in some patients with ischemic heart disease [1, 7, 10, 11, 15–17]. Suwa et al. reported that ILVT was no longer induced after surgical resection of FTs [18]. However, the anatomical and electrophysiological characteristics of FTs related to ventricular arrhythmias have not been well clarified.

**Figure 6:**
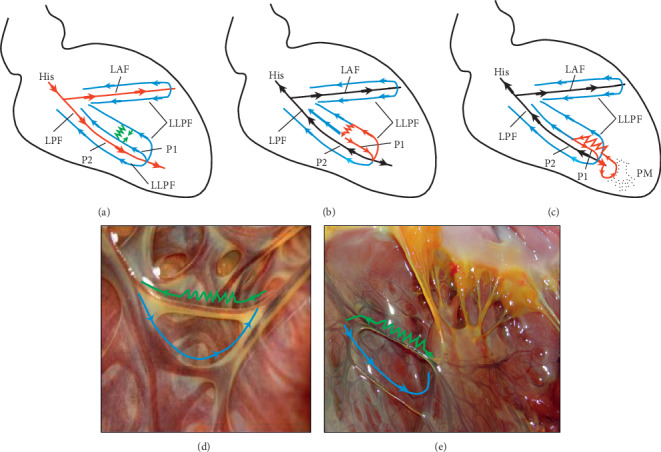
The schematics of our hypothesis about the possible reentrant circuits of idiopathic left fascicular ventricular tachycardia. (a) The anatomical structure and conduction pattern of the Purkinje network during sinus rhythm. During sinus rhythm, the main branch of His-Purkinje system (the apical hierarchy) exhibited antegrade activation sequence (P2, red lines and arrows) and then retrogradely activated the Purkinje network on the basal hierarchy (P1, blue lines and arrows). The green line and arrows represented the tiny FT between the Purkinje network on the same basal hierarchy or different hierarchies. (b) During VT, the reentrant circuit was around the Purkinje fiber network, which might be connected by the tiny FT as a slow conduction zone (red lines and arrows). To note, the left posterior fascicle (P2) was a bystander of the reentrant circuit. (c) During VT in rare cases, the slow conduction zone was from a longer FT connecting the Purkinje network and the papillary muscles (red lines and arrows). (d) The possible anatomical basis of idiopathic left fascicular VT in (b) (e) The possible anatomical basis of idiopathic left fascicular VT in (c) LPF, left posterior fascicle; LAF, left anterior fascicle; LLPF, lower or basal hierarchy Purkinje fibers; PM, papillary muscle.

Because of the correlation between Purkinje fibers and FTs, FTs may be involved in a variety of ventricular arrhythmias. For example, the underlying mechanism of left posterior fascicular ventricular tachycardia (LPF-VT) has been demonstrated to be reentry involving the left ventricular conduction system [1, 19–21]. However, the exact reentrant circuit, especially the substrate of the slow conduction zone, remains somewhat unclear. In few cases with LPF-VT, a FT connecting the septal portion of the LPF and the papillary muscles was identified to be one part of the reentrant circuit [10, 11]. However, in most cases with LPF-VT, considerable FTs could not be detected. In the present study, tiny FTs were found connecting the adjacent Purkinje network on the endocardium. We speculated that the tiny FT might play an important role in the reentrant circuit of LPF-VT. The sharp turn or anisotropic conduction between FT and the adjacent Purkinje network might be the substrate of slow conduction zone (Figure 6).

### 4.5. Limitations

There were several limitations in the present study. Firstly, this was an in vitro animal study, and the electrophysiologic properties might be different to some extent from those of an in vivo or in a Langendorff perfusion heart model. Secondly, the duration available for visual mapping of the electrophysiological characteristics of Purkinje fibers in detail was limited. Thirdly, the characteristics of smaller and tiny FTs and their response to verapamil or catecholamine have not been well clarified in this study. Further research should be carried out on the histological and electrophysiological characteristics of the smaller and tiny FTs.

## 5. Conclusions

The Purkinje fibers are widely distributed in canine FTs, including smaller and tiny FTs. The Purkinje fibers contained in the FTs extend from the proximal to the distal end, anatomically and electrophysiologically connecting the endocardial Purkinje fiber system, which might be the important substrate for ventricular arrhythmias.

## Data Availability

The statistical and image data used to support the findings of this study are included within the article.
